# Dynamically Optimizing Network Structure Based on Synaptic Pruning in the Brain

**DOI:** 10.3389/fnsys.2021.620558

**Published:** 2021-06-04

**Authors:** Feifei Zhao, Yi Zeng

**Affiliations:** ^1^Research Center for Brain-inspired Intelligence, Institute of Automation, Chinese Academy of Sciences, Beijing, China; ^2^Institute of Automation, Chinese Academy of Sciences, Beijing, China; ^3^National Laboratory of Pattern Recognition, Institute of Automation, Chinese Academy of Sciences, Beijing, China; ^4^Center for Excellence in Brain Science and Intelligence Technology, Chinese Academy of Sciences, Shanghai, China; ^5^University of Chinese Academy of Sciences, Beijing, China

**Keywords:** synaptic pruning, developmental neural network, optimizing network structure, accelerating learning, compressing network

## Abstract

Most neural networks need to predefine the network architecture empirically, which may cause over-fitting or under-fitting. Besides, a large number of parameters in a fully connected network leads to the prohibitively expensive computational cost and storage overhead, which makes the model hard to be deployed on mobile devices. Dynamically optimizing the network architecture by pruning unused synapses is a promising technique for solving this problem. Most existing pruning methods focus on reducing the redundancy of deep convolutional neural networks by pruning unimportant filters or weights, at the cost of accuracy drop. In this paper, we propose an effective brain-inspired synaptic pruning method to dynamically modulate the network architecture and simultaneously improve network performance. The proposed model is biologically inspired as it dynamically eliminates redundant connections based on the synaptic pruning rules used during the brain's development. Connections are pruned if they are not activated or less activated multiple times consecutively. Extensive experiments demonstrate the effectiveness of our method on classification tasks of different complexity with the MNIST, Fashion MNIST, and CIFAR-10 datasets. Experimental results reveal that even for a compact network, the proposed method can also remove up to 59–90% of the connections, with relative improvement in learning speed and accuracy.

## 1. Introduction

Deep Neural Network (DNNs) have achieved state-of-the-art performance for various machine learning tasks, including image classification (Krizhevsky et al., 2012; He et al., 2015; Simonyan and Zisserman, 2015), face recognition (Lawrence et al., 1997), video prediction (Deng et al., 2013), and speech recognition (Hinton et al., 2012; Abdel-Hamid et al., 2014). In spite of their superior performance, the complex network architectures lead to a significant increase in the computation and parameter storage costs, which limits their deployment on resource-constrained devices. Besides, excessive number of parameters will lead to over-fitting. Dynamically optimizing a fully connected network by removing redundant connections is a promising approach to compress network and avoid over-fitting.

To realize the dynamic modulation of a network structure, two key issues need to be resolved. First, which synaptic connections in the network are redundant? Second, when should redundant synaptic connections be removed? We take inspiration from the highly efficient and complex central nervous system, which is a complex neural network and is modulated and pruned during development. Throughout the developmental process of childhood and adolescence, synaptic overgrowth followed by the selective elimination of redundant synapses (Montagu, 1964; Chechik et al., 1999). The activity of the synapses determines whether they will be eliminated or retained. When learning tasks, repeated use will strengthen the synapses, while the rarely used synapses will become weaker and likely to be eliminated (Pascual-Leone et al., 2005; Mangina and Sokolov, 2006; Johnston et al., 2009). As a result, redundant synapses are pruned from the brain, leaving only the most important synapses. This brain pruning mechanism inspired some minimal-value deletion methods. They prune the synapses with weights below a threshold (Chechik et al., 1998a,b; Han et al., 2015). However, these methods are somewhat arbitrary because they eliminate some synapses whose weights are incidentally below the threshold. Moreover, the thresholds need to be carefully defined for different conditions.

In this paper, we propose a brain-inspired synaptic pruning (BSP) algorithm based on the synaptic pruning mechanism in the human brain. Our method prunes unimportant synapses that have been hardly used for consecutive multiple times. In this way, during the learning process, the proposed method can effectively modulate neural network architecture by pruning redundant synapses while retaining effective synapses. In order to verify the generality of our method, we test it on classification tasks of different complexity with the MNIST, Fashion MNIST, and CIFAR-10 datasets. When applied to the networks with different sizes and different numbers of training samples, our method validates its strengths and effectiveness. Experimental results demonstrate that BSP can significantly compress the network. More importantly, compared with the initial network and the dropout network, the pruned network has similar test accuracy, but the learning speed is much faster.

## 2. Related Work

This section introduces some related works on optimizing network architecture. Pruning network has been widely studied in recent years. Minimal-value deletion pruned all synapses whose weights are below a threshold (Chechik et al., 1998a,b; Han et al., 2015). The experimental results showed that the pruned network can be significantly compressed without affecting accuracy. However, this method may prune some useful synapses whose weights are incidentally below the threshold. Other works focus on designing appropriate criteria to evaluate the importance of synapses so that the least important ones are pruned. Molchanov et al. (2017) considered the l2-norm of the kernel weights, as well as the mean, standard deviation, and percentage activation of the feature map. They also used mutual information between activations and predictions as an evaluation criterion. A first-degree Taylor expansion method was proposed in Molchanov et al. (2017) to evaluate the importance of synapses. LeCun et al. (1990) and Hassibi and Stork (1993) focused on the second-order term of a Taylor expansion and calculated the importance of synapses using a diagonal Hessian matrix. He et al. (2019) proposed a filter pruning method based on the geometric median to prune the most replaceable filters containing redundant information. Yu et al. (2018) proposed the neuron importance score propagation (NISP) algorithm, which propagates the importance scores of final responses to every neuron in the network. Then, the convolutional neural network was pruned by removing neurons with the least importance. Li et al. (2016) removed the filters with relatively low weights together with their connecting feature maps. He et al. (2018) proposed a soft pruning method that enables the pruned filters to be updated when training the model after pruning. These methods have little biological plausibility and mainly focus on the regularization of the neural network. In addition, improving the regularization is often at the expense of accuracy.

Dropout (Srivastava et al., 2014) is widely used to prevent over-fitting. In dropout, each neuron is probabilistically dropped during training but can return during inference. There is no reduction in the complexity of a network with this method. DropConnect (Wan et al., 2013) randomly set a subset of weights within a neural network to zero, which helped in regularizing the network. In some cases, it outperformed dropout but was slower at learning than the initial network and the dropout network. MeProp (Sun et al., 2017) updated a small portion of the parameters during each backpropagation step. These methods do not essentially change the structure of the network.

Some methods use evolutionary strategies to optimize a network structure dynamically. Evolutionary artificial neural networks optimize network weights and network structure simultaneously. Some parameters related to network structure are encoded into the genome, which are optimized by an evolutionary strategy. An evolutionary strategy evaluates the performance of a network with a fitness function. Such functions usually include classification accuracy (e.g., the reciprocal of the error or the mean squared error Angeline et al., 1994; Yao and Liu, 1996 or the cross-entropy error Park and Abusalah, 1997) and the network scale (e.g., the number of neurons or connections Vonk et al., 1995; Ioan et al., 2004). After several iterations, an evolutionary artificial neural network can find the optimal network structure. Zhao et al. (2017) proposed an evolutionary optimization method that prunes a network to an appropriate network topology. These methods focus on optimizing the network structure to attain the best balance between network complexity and test accuracy. However, the evolution process is time-consuming, and these methods have some randomness, which may result in significant detours.

In summary, existing network optimization methods rarely considered the neural development of a biological brain. The dynamic development in the brain enables a very small network to complete complex tasks. This paper develops a dynamic synaptic pruning method inspired by the brain's pruning mechanism. Our experimental results on different classification tasks demonstrate that the proposed method can improve the test accuracy and convergence speed, even when the initial network is compressed to a very small size.

## 3. Methods

In this section, we will introduce BSP method in detail. We first present the overall framework of BSP method. Next, a more detailed pruning strategy would be presented. Finally, we will show the implementation details for a three-layer fully connected neural network.

**Figure 1:**
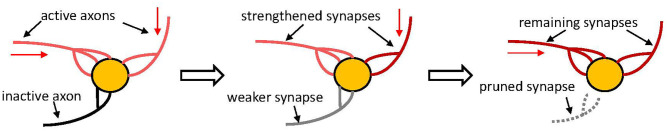
The synaptic pruning strategy of BSP algorithm.

Our synaptic pruning method is inspired from the developmental process in the human brain. When learning tasks, a proportion of the synapses are strengthened while a proportion of them are weakened (Hayashi-Takagi et al., 2015). Synapses that are frequently used will be strengthened and maintained, while weaker synapses that have not been activated for a long time will be shrunk and pruned (Sanes and Lichtman, 1999; Rao et al., 2012). The goal of synaptic pruning is to discard the less used or redundant synapses. In this paper, we first establish a non-trained three-layer ANN as the initial network and ensure that the network is sufficiently complex. Then, during training, we iteratively prune unimportant synapses and update the weights of remaining synapses through back-propagation. As depicted in Figure 1, synapses that are continually weaker will be pruned in each epoch.

**Figure 2:**
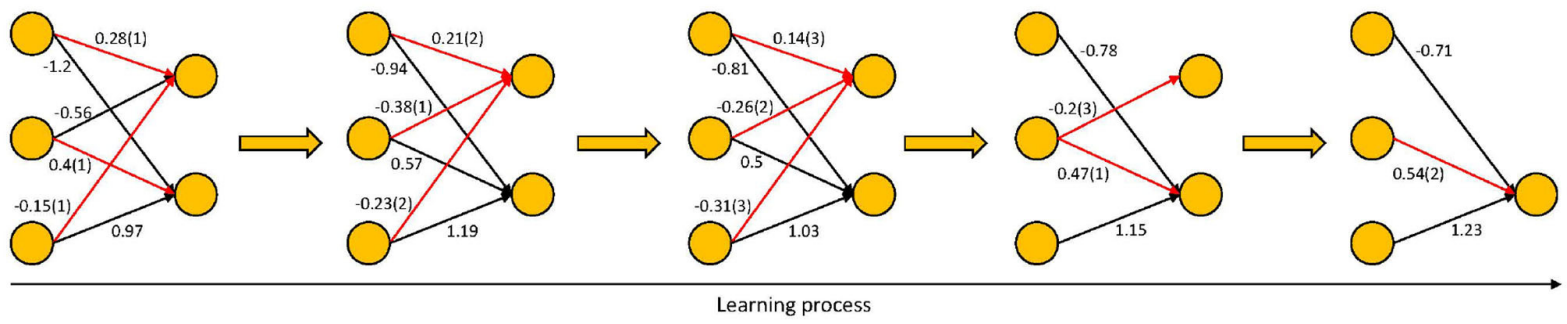
The detailed pruning process of BSP algorithm. The red connections represent the candidate pruned synapses.

The detailed pruning strategy has the following three steps:

(1) Evaluate the importance of connections and select candidate pruned synapses. We measure the relative importance of a connection by its absolute weight. In each iteration, we select synapses with smaller absolute weights as candidate pruning synapses. These synapses have little effect on the final output and could be considered as weaker synapses. The candidate pruning synapses are determined by the pruning rate rather than the threshold. In this way, pruning is fairer and more adaptive. The red connections in Figure 2 represent the candidate pruned synapses.
(2) Calculate the number of consecutive times that a synapse is a candidate to be pruned. If a synapse always belongs to the weaker ones, the number of consecutive times will be large, indicating that the synapse is unimportant. If a synapse is sometimes used, we will keep it and monitor it. In Figure 2, the values in parentheses represent the number of consecutive times that the connections have belonged to the set of candidate pruned synapses.
(3) Prune the synapses whose number of consecutive times exceed the threshold. Directly removing the candidate pruned synapses may result in a sharply and potentially irrecoverable drop in accuracy. Only prune the synapses that have not been used for a long time can ensure that the pruned synapses are redundant. In Figure 2, the threshold is 3, so pruning starts on the fourth epoch. Pruning permanently eliminates unused synapses and reduces the network complexity.

Next, we describe the implementation detail on a three-layer fully connected neural network. First, we define some parameters used by BSP algorithm. The core parameters are the pruning ratio *p*_*r*_ and the threshold for the number of consecutive times *p*_*c*_. Let *N* be the number of synapses in the initial network, *N*_*s*_ the remaining number of synapses in the current iteration, and *N*_*c*_ the number of candidate pruned synapses, where *N*_*c*_ = *N*_*s*_ × *p*_*r*_. The set of candidate pruned synapses is *C*_*w*_. The set of pruned synapses is *P*_*w*_, and the number of pruned synapses is *N*_*p*_.

In this paper, we verify the performance of BSP algorithm on classification tasks with different complexity. The parameters *p*_*r*_ and *p*_*c*_ are dynamically modulated for different conditions. Suppose the initial number of neurons in the hidden layer is *N*_neu_, and the number of training samples is *N*_sam_. Then, *p*_*r*_ increases with an increase of *N*_neu_. The larger *N*_sam_ is, the smaller the *p*_*r*_ will be. Thus, we define *p*_*r*_ as follows:

(1) $$\begin{array}{r} p_{r}=\frac{\alpha \times \log_{e}\left(N_{\text{neu}}\right)}{\log_{e}{\left(\beta \times \left(N_{\text{sam}}/B\right)\right)}^{2}} \end{array}$$

*p*_*c*_ decreases with an increase of *N*_neu_, but increases with *N*_sam_. Thus, we define *p*_*c*_ as as follows:

(2) $$\begin{array}{r} p_{c}=2^{N/\left(N-N_{p}\right)}\times \frac{A}{N_{\text{neu}}+\mu }\times \left[{\left[\log_{e}\left(\beta \times \frac{N_{\text{sam}}}{B}\right)\right]}^{2}+1\right] \end{array}$$

The constants in Equations (1) and (2) are carefully defined based on our experience: α = 0.048, β = 50, μ = 146, *A* = 1244, and *B* = 60000. Here, *p*_*c*_ changes exponentially with the number of pruned synapses, which prevents the network from being over-pruned. If the number of remaining synapses is too small, *p*_*c*_ will automatically increase to limit pruning.

In this paper, the weights of the pruned synapses are set to zero during both training and testing phases. That is, the pruned synapses have no effect on the later feedforward process and will not be updated during the feedback process. Consider the *j*th neuron in the hidden layer. *x*_*i*_ is the input to neuron *h*_*j*_ in the hidden layer, *y*_*j*_ denotes the output of neuron *h*_*j*_, and *w*_*ij*_ and *b*_*j*_ are the weight and bias, respectively. If *P*_*w*_ is the set of pruned synapses, then the feedforward and feedback functions are as follows:

(3) $$\begin{array}{r} y_{i}=f\left(\overset{n}{\underset{i=1}{\sum }}p\left(w_{ij}\right)w_{ij}x_{i}+b_{j}\right) \end{array}$$

(4) $$\begin{array}{r} w_{ij}=p\left(w_{ij}\right)\times \left(w_{ij}-\eta \frac{\partial E}{\partial w_{ij}}\right) \end{array}$$

where *f* is the activation function, *E* is any loss function (for example, the mean squared error function), and η is the learning rate. Function *p* is calculated with

(5) $$\begin{array}{r} p\left(w_{ij}\right)=\{\begin{array}{cc} 1, & w_{ij}\notin P_{w}\\ 0, & w_{ij}\in P_{w} \end{array} \end{array}$$

If the synapse belongs to the set of pruned synapses *P*_*w*_, it will not be used or updated. The detailed framework of BSP algorithm is shown in Algorithm 1.

**Algorithm 1:** The BSP algorithm.

**Input:** Initial fully connected neural network with enough complexity; **Output:** Pruned neural network;
1: Initialize *C*_*w*_ = [ ], *P*_*w*_ = [ ], *N*_*c*_ = *N*_*p*_ = 0;
2: Calculate *p*_*r*_, *p*_*c*_ according to Equation (1) and (2);
3: **for** iteration **do**
4: Forward computation from Equation (3);
5: Backpropagation computation from Equation (4) and (5);
6: Choosing the candidate pruned synapses *C*_*w*_ = {*w*_1_, *w*_2_, …, *w*_*N*_*c*__};
7: **for** each *w*_*i*_ ∈ *C*_*w*_ **do**
8: Counting the number of consecutive times $w_{i}^{c}$ that connection *w*_*i*_ belongs to *C*_*w*_;
9: **if** $w_{i}^{c}>p_{c}$ **then**
10: *P*_*w*_ = *P*_*w*_⋃*w*_*i*_;
11: **end if**
12: **end for**
13: Pruning the least important synapses *P*_*w*_;
14: **end for**

## 4. Results

We evaluate our method on different tasks, including different datasets, training samples with different complexities, and different network scales. Our method is applied to a three-layer ANN with one input layer, one output layer, and one hidden layer. The activation function for neurons in the input and hidden layers is the sigmoid function. We use the softmax activation function in the output layer. The learning rate is 0.1, and the number of iterations is 500.

The goal of this work is to explore whether BSP algorithm can improve the classification accuracy and convergence speed even when many connections are discarded. To verify the generalization of our method, we test it on classification tasks of different complexity with the MNIST, Fashion MNIST, and CIFAR-10 datasets. We compare our method with the dropout method, which is an effective method for avoiding over-fitting. We set the dropout rates with the best performance of the dropout network. We evaluate our method using the network compression, the improvement in classification speed and test accuracy compared with the initial neural network and the network with dropout. The network compression is the ratio of the number of zero weights in BSP network to the number of connections. The improvement in learning speed *L* is calculated as follows:

(6) $$\begin{array}{r} L=\frac{T_{i}^{c}}{T_{i}^{b}},\;\text{s.t.}\;a_{i}^{b}=a_{i}^{c},\;i=\underset{i}{\max}|T_{i}^{b}-T_{i}^{c}| \end{array}$$

where the vectors *T*^*b*^ and *T*^*c*^ represent all the times at which BSP algorithm and the compared method (either the initial network or the dropout network, respectively) have the same accuracy. For any *i*th element in *T*^*b*^ and *T*^*c*^, the accuracy of BSP algorithm $a_{i}^{b}$ is equal to the accuracy of the compared method $a_{i}^{c}$. We then find the index *i* with the maximal difference between the learning times for BSP algorithm $T_{i}^{b}$ and the compared method $T_{i}^{c}$.

### 4.1. Experiments on MNIST

The MNIST dataset contains 10 classes of handwritten digits from 0 to 9, with 60,000 training samples and 10,000 test samples (Lecun et al., 1998). Each sample is represented by a 28 × 28 digital image. The initial ANN has 784 neurons in its input layer and 10 in its output layer. To verify the general performance for the MNIST dataset, we use 10, 100, and 500 neurons in the hidden layer at the beginning of the ANN training. We train the models on either 1,200 or 60,000 training data points. The dropout rates for the MNIST dataset are listed in Table 1. We do not compare to the dropout method with 10 neurons because the dropout could not improve performance when there are only 10 neurons in the hidden layer.

**Table 1:** Dropout rates for different numbers of training samples and network sizes for the MNIST dataset.

**Number of samples**	**10 neurons**	**100 neurons**	**500 neurons**
60,000	0	0.3	0.4
1,200	0	0.4	0.6

#### Results for 60,000 Training Samples

The test accuracy, improvement in learning speed, and network compression are compared in Table 2. The first three rows show the test accuracies of the initial network *A*_init_, the dropout network *A*_dropout_, and our method *A*_BSP_. Our method outperforms the initial and dropout networks in all cases. With 10 neurons in the hidden layer, dropout could not improve the accuracy while our method improves the accuracy from 91.76 to 92.32%. The next rows show the improvement in learning speed compared to the initial network *L*_BSP-init_ and the dropout network *L*_BSP-dropout_. The BSP method can accelerate learning and improve test accuracy at the same time compared with the initial and dropout networks. The network compression is shown in the final row. We can conclude that our method compresses the network significantly in all cases.

**Table 2:** Comparison of test accuracy, improvement in learning speed, and network compression for 60,000 MNIST training samples.

	**10 neurons**	**100 neurons**	**500 neurons**
*A*_init_(%)	91.76	95.61	96.15
*A*_dropout_(%)	–	95.90 **(+0.29)**	96.77 **(+0.62)**
*A*_BSP_(%)	92.32 **(+0.56)**	95.94 **(+0.33)**	96.84 **(+0.69)**
*L*_BSP-init_	1.2188	2.76	1.67
*L*_BSP-dropout_	–	1.92	2.71
**Network compression**	**59.26%**	**83.38%**	**87.96%**

*The bold values mean the improvement of accuracy compared to the initial network*.

**Figure 3:**
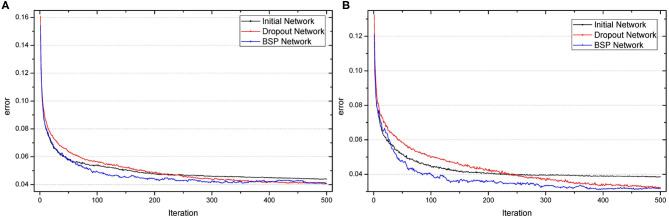
Test error as a function of the number of iterations when the number of neurons in the hidden layer was 100 **(A)** or 500 **(B)** for 60,000 MNIST training samples.

In summary, BSP algorithm improves accuracy and learning speed compared with the initial and dropout networks. Moreover, the networks can be significantly compressed. Figure 3 shows the change in the error during the iteration. It is obvious that our method has the quickest learning speed compared with the initial and dropout networks. Besides, BSP algorithm improves performance with faster learning speed whereas dropout slows down the learning speed.

#### Results for 1,200 Training Samples

A network with good generalization should work well on both large and small training sets. For a small task with 1,200 training samples, comparisons of the test accuracy, improvement in learning speed, and network compression are shown in Table 3. Compared with the initial network, our method has better accuracy to some extent. When the network is too small (with 10 neurons in the hidden layer), dropout cannot improve classification performance compared to the initial network, whereas our method works. With 500 neurons in the hidden layer, the accuracy for our method is not better than that of the dropout method, but it is still better than the initial network. This indicates that our method can avoid over-fitting to some extent. Note that *L*_BSP-init_ and *L*_BSP-dropout_ are always larger than 1, which indicates that our method has a faster learning speed compared with the initial and dropout networks. Finally, our method can significantly compress the network and reduce the amount of storage space needed.

**Table 3:** Comparison of test accuracy, improvement in learning speed, and network compression for 1,200 MNIST training samples.

	**10 neurons**	**100 neurons**	**500 neurons**
*A*_init_(%)	82.55	85.05	86.53
*A*_dropout_(%)	–	87.43 **(+2.38)**	88.92 **(+2.39)**
*A*_BSP_(%)	82.74 **(+0.19)**	87.5 **(+2.45)**	87.69 **(+1.16)**
*L*_BSP-init_	1.0357	3.83	5.47
*L*_BSP-dropout_	–	1.6	1.33
**Network compression**	**81.85%**	**85.95%**	**90.31%**

*The bold values mean the improvement of accuracy compared to the initial network*.

In summary, for both 1,200 and 60,000 MNIST training samples, our method can significantly compress the network and improve the learning speed compared with the initial and dropout networks. The BSP algorithm has better test accuracy than the initial network and comparable test accuracy with the dropout network.

### 4.2. Experiments on Fashion MNIST

The Fashion MNIST classification dataset contains 10 classes: T-shirts, trousers, pullovers, dresses, coats, sandals, shirts, sneakers, bags, and ankle boots. It has 28 × 28 grayscale images of 60,000 training samples and 10,000 test samples (Xiao et al., 2017). To verify the general performance on the Fashion MNIST dataset, we use 10, 100, and 500 neurons in the hidden layer at the beginning of the ANN training. We train the models on either 1,200 or 60,000 training data points. The dropout rates used are the same as that for the MNIST dataset. The detailed comparisons are as follows.

#### Results for 60,000 Training Samples

The test accuracy, improvement in learning speed, and network compression are compared in Table 4. Our method can improve the test accuracy and learning speed compared with the initial network. For 100 and 500 neurons in the hidden layer, our method could not exceed the accuracy of the dropout method, but can accelerate the learning. In summary, BSP algorithm can improve learning speed while significantly compressing the network, and avoiding over-fitting, to some extent.

**Table 4:** Comparison of test accuracy, improvement in learning speed, and network compression for 60,000 Fashion MNIST training samples.

	**10 neurons**	**100 neurons**	**500 neurons**
*A*_init_(%)	83.73	86.56	87.78
*A*_dropout_(%)	–	88.34 **(+1.78)**	89.08 **(+1.3)**
*A*_BSP_(%)	84.33 **(+0.6)**	86.91 **(+0.35)**	88.4 **(+0.62)**
*L*_BSP-init_	1.0526	1.14	2.12
*L*_BSP-dropout_	–	2.19	2.65
**Network compression**	**61.49%**	**83.08%**	**87.87%**

*The bold values mean the improvement of accuracy compared to the initial network*.

#### Results for 1,200 Training Samples

Table 5 compares the test accuracy, improvement in learning speed, and network compression for 1,200 training samples. With 10 neurons in the hidden layer, the accuracy of BSP algorithm is lower by 0.7 percentage points compared with the initial network, while the learning speed is improved and the network is compressed by 81.74%. For the network with 100 and 500 neurons in the hidden layer, BSP algorithm can improve the test accuracy compared to the initial network and has comparable accuracy with the dropout method. Besides, our method can significantly accelerate learning and compress the network.

**Table 5:** Comparison of test accuracy, improvement in learning speed, and network compression for 1,200 Fashion MNIST training samples.

	**10 neurons**	**100 neurons**	**500 neurons**
*A*_init_ (%)	76.15	77.97	79.13
*A*_dropout_ (%)	–	79.87 **(+1.9)**	80.8 **(+1.67)**
*A*_BSP_ (%)	75.5	79.25 **(+1.28)**	79.75 **(+0.62)**
*L*_BSP-init_	1.4174	2.56	1.95
*L*_BSP-dropout_	–	1.5	1.67
**Network compression**	**81.74%**	**85.72%**	**89.61%**

*The bold values mean the improvement of accuracy compared to the initial network*.

### 4.3. Experiments on CIFAR-10

The CIFAR-10 dataset consists of 50,000 training images and 10,000 test images, which can be divided into 10 categories: airplanes, automobiles, birds, cats, deer, dogs, frogs, horses, ships, and trucks (Krizhevsky, 2009). For networks with 10, 100, and 500 neurons in the hidden layer, the dropout rates are equal to 0.3. Table 6 compares the test accuracy, improvement in learning speed, and network compression. For a network with 10 neurons in the hidden layer, BSP algorithm has a higher test accuracy compared with the initial and the dropout networks. For the networks with 100 and 500 neurons in the hidden layer, BSP algorithm has a better test accuracy than the initial network but is inferior to the dropout network. This indicates that BSP algorithm can avoid over-fitting, to some extent. Besides, BSP algorithm can accelerate the learning and compress the network compared with both the other networks.

**Table 6:** Comparison of test accuracy, improvement in learning speed, and network compression for CIFAR-10 training samples.

	**10 neurons**	**100 neurons**	**500 neurons**
*A*_init_ (%)	37.6	46.81	51.62
*A*_dropout_ (%)	38.23 **(+0.63)**	51.63 **(+4.82)**	56.52 **(+4.9)**
*A*_BSP_ (%)	39.08 **(+1.48)**	48.68 **(+1.87)**	53.55 **(+1.93)**
*L*_BSP-init_	1.84	1.78	1.46
*L*_BSP-dropout_	4.5	1.33	1.57
**Network compression**	**68.75%**	**83.33%**	**87.91%**

*The bold values mean the improvement of accuracy compared to the initial network*.

### 4.4. Effect on Sparsity

**Figure 4:**
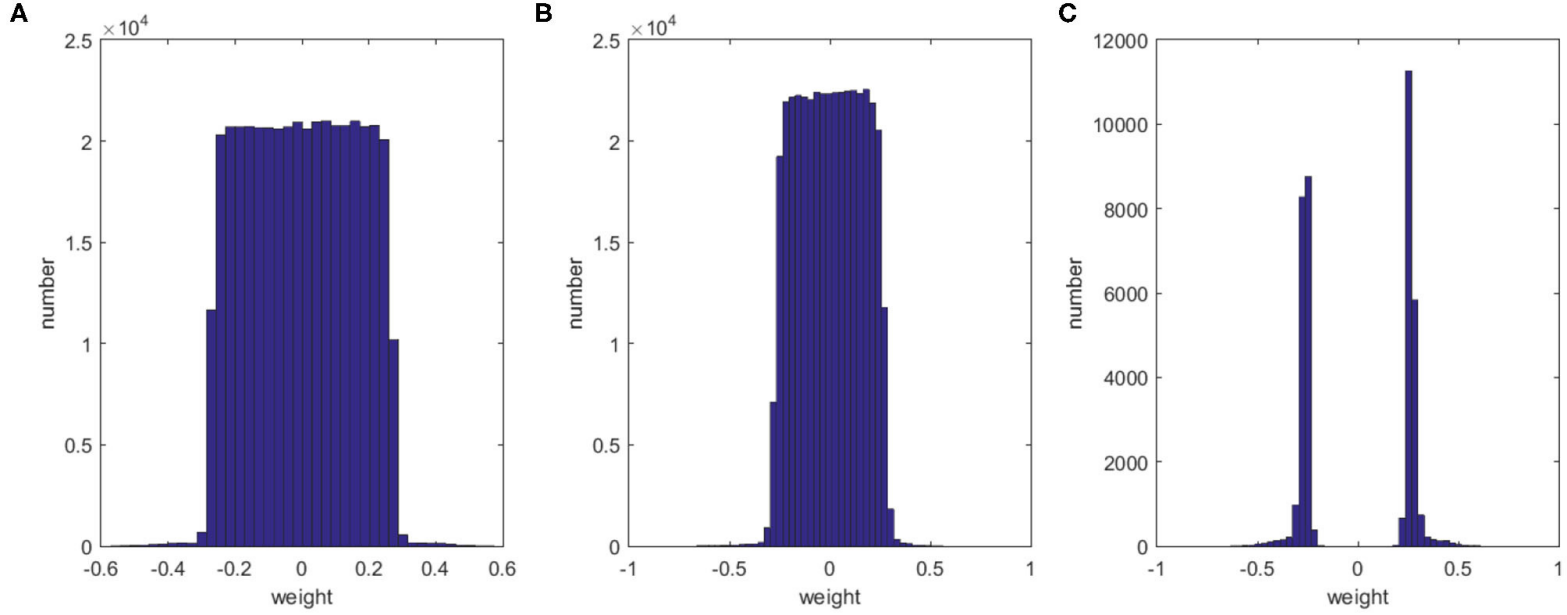
Histograms showing the distribution of weights for the initial network **(A)**, the dropout network **(B)**, and our method **(C)**.

In this section, we discuss the effect of our method on the sparsity of the network structure. Taking 1,200 training samples from the MNIST dataset and training with 500 hidden neurons as an example, the histograms in Figure 4 show the distributions of the weights for the initial network, the dropout network, and our method after 500 iterations. Clearly, our method has fewer synapses and the weights are more sparse than those of the other networks. Though dropout randomly inactivates some neurons during training, this has only a small impact on the weights of the connections. Our method can significantly compress the network, leaving only 9.69% synapses from the initial network while still improving the performance and learning speed.

## 5. Conclusion

Inspired by the synaptic pruning mechanism during the brain's development, this paper proposes a BSP algorithm that adaptively modulates a neural network architecture by pruning redundant synapses during learning. The BSP algorithm prunes consecutively unused synapses and retains only the important ones. To assess the performance of our method, we test it on classification tasks of different complexity with different datasets. Our experimental results show that the pruned network can be significantly compressed, and more importantly, the pruned network has a similar test accuracy but much quicker learning speed compared with the initial and dropout networks. In summary, our method shows three improvements for an ANN: avoiding over-fitting, compressing the network, and improving the learning speed.

## Data Availability Statement

Publicly available datasets were analyzed in this study. This data can be found here: MNIST dataset: http://yann.lecun.com/exdb/mnist/, Fashion MNIST dataset: https://github.com/zalandoresearch/fashion-mnist, CIFAR-10 dataset: https://www.cs.toronto.edu/~kriz/cifar.html.

## Author Contributions

FZ and YZ designed the study, performed the experiments, and wrote the manuscript. All authors contributed to the article and approved the submitted version.

## Conflict of Interest

The authors declare that the research was conducted in the absence of any commercial or financial relationships that could be construed as a potential conflict of interest.
